# Comparison of the systemic and pulmonary inflammatory response to endotoxin of neutropenic and non-neutropenic rats

**DOI:** 10.1186/1476-9255-4-7

**Published:** 2007-03-30

**Authors:** Sabrina M Heidemann, Maria Glibetic

**Affiliations:** 1 Department of Pediatric Critical Care Medicine and Clinical Pharmacology, Wayne State University, Detroit, MI, USA; 2 Children's Hospital of Michigan, 3901 Beaubien, Detroit, MI 48201, USA

## Abstract

**Background:**

Neutrophil infiltration commonly occurs in acute lung injury and may be partly responsible for the inflammatory response. However, acute lung injury still occurs in the neutropenic host. The objectives of this study are to determine if inflammation and acute lung injury are worse in neutropenic versus the normal host after endotoxemia.

**Methods:**

Rats were divided into four groups: 1) control, 2) neutropenic, 3) endotoxemic and 4) endotoxemic and neutropenic. Tumor necrosis factor (TNF)-α and macrophage inflammatory protein (MIP-2) were measured in the blood, lung lavage and for mRNA in the lung. Arterial blood gases were measured to determine the alveolar-arterial oxygen gradient which reflects on lung injury.

**Results:**

In endotoxemia, the neutropenic rats had lower plasma TNF-α (116 ± 73 vs. 202 ± 31 pg/ml) and higher plasma MIP-2 (26.8 + 11.9 vs. 15.6 + 6.9 ng/ml) when compared to non-neutropenic rats. The endotoxemic, neutropenic rats had worse lung injury than the endotoxemic, non-neutropenic rats as shown by increase in the alveolar-arterial oxygen gradient (24 ± 5 vs. 12 ± 9 torr). However, lavage concentrations of TNF-α and MIP-2 were similar in both groups.

**Conclusion:**

Neutrophils may regulate TNF-α and MIP-2 production in endotoxemia. The elevation in plasma MIP-2 in the endotoxemic, neutropenic rat may be secondary to the lack of a neutrophil response to inhibit production or release of MIP-2. In endotoxemia, the severe lung injury observed in neutropenic rats does not depend on TNF-α or MIP-2 produced in the lung.

## Background

Sepsis is a leading cause of morbidity and mortality in the intensive care unit. Improvements in the treatment of cancer have led to a growing population of immunocompromised patients with longer survival times and a propensity to develop sepsis and acute respiratory distress syndrome (ARDS) [1,2]. It is important to study the role of neutrophils in sepsis in order to better understand the effect of neutropenia on the inflammatory response in sepsis. Different treatment modalities may be necessary in the neutropenic versus the non-neutropenic host.

Neutropenia is commonly induced by cyclophosphamide in cancer-stricken patients. In these patients, white cells are markedly diminished in number but not absent. It is known that cyclophosphamide has effects on other cells in the body [3-5]. This drug may decrease the activity of lymphoid cells and macrophages in the spleen [3,4]. Cyclophosphamide may also modulate CD4+ T cells into a Th2 phenotype and cause a decrease IFN-gamma production as in patients with multiple sclerosis [5]. However, in spite of the numerous cellular effects of cyclophosphamide, in a study of endotoxemia-associated acute lung injury, depletion of neutrophils by cyclophosphamide or anti-neutrophil antibodies showed no difference in lung injury as shown by lung edema or inflammation as demonstrated by similar amounts of the transcription factor, nuclear factor kappa B [6]. Cyclophosphamide can be used to induce neutropenia because of its widespread use in treatment regimens and the fact that the effect is similar to anti-neutrophil antibodies when studying acute lung injury secondary to endotoxemia.

The immune response to sepsis has been widely studied in the immunocompetent host. Sepsis leads to early release of the cytokines; tumor necrosis factor (TNF)-α and interleukin (IL)-1β which are primarily produced by macrophages [7]. Cytokines are low molecular weight proteins (<30 kd) that are responsible for intercellular signaling [8]. After TNF-α and IL-1β are released, their signals are amplified many fold, leading to the activation of the inflammatory cascade and consequently, inflammation. The role of the neutrophil in producing or modulating this initial cytokine response to sepsis has not been well studied [6].

Recruitment of neutrophils in the lung for example, depends on communication between endothelial, stromal, parenchymal cells and the infiltrating neutrophils [9]. These initial events are mediated by the early production of cytokines, specialized cytokines called chemokines and cell adhesion molecules [9]. Chemokines belong to a superfamily of cytokines that promote chemotaxis of leukocytes to areas of infection, tissue injury or neoplasia [10]. Four subfamilies of chemokines are categorized based on the spacing of the first two cysteine residues and are designated as C, C-C, C-X-C, C-X_3_-C. In general, the C-X-C group is responsible for neutrophil chemotaxis and activation [11,12]. The most studied C-X-C chemokine in humans is IL-8. No murine homolog for IL-8 has been discovered but macrophage inflammatory protein (MIP)-2 is one of its functional homologs [13]. In sepsis, the development of acute lung injury may be secondary to activation of the inflammatory response.

Sepsis syndrome is the single most common risk factor associated with the development of acute respiratory distress syndrome (ARDS) [14]. Inflammatory mediators that may be produced as a result of sepsis are felt to play a central role in the development of ARDS. Migration of neutrophils to the lungs and disruption of the alveolar capillary membrane characterizes early ARDS [7,15]. Mechanisms involved in recruiting neutrophils into the lung have not been well established but are probably dependent on chemotactic factors produced in the lung [7,15].

The C-X-C chemokine, macrophage inflammatory protein (MIP-2), contributes to increasing neutrophil chemotaxis in murine acute lung injury [16-23]. Lipopolysaccharide, TNF-α and/or IL-1β have been reported to stimulate the production of these cytokines in the lung [19,22]. In the neutropenic host, neutrophil adhesion and chemotaxis may be abnormal due to insufficient production of chemokines or disruption of at feedback mechanisms. Regulation of the inflammatory response by neutrophils is unknown and has not been well studied.

The first objective of this study is to determine if TNF-α, IL-1β and MIP-2 concentrations will be lower in the systemic circulation of neutropenic, endotoxemic rats compared to non-neutropenic, endotoxemic rats. The second objective is to ascertain if acute lung injury will be worse secondary to the increased production of TNF-α, IL-1β and MIP-2 in the lungs of the non-neutropenic, endotoxemic rats versus the neutropenic endotoxemic rats.

## Materials and methods

This study was approved by the Animal Investigation Committee at Wayne State University and performed in accordance with the NIH guidelines for the use of animals in research. Male Sprague-Dawley rats weighing 250–400 g were divided into one of four groups: 1) normal control (n = 6), 2) neutropenic control (n = 6), 3) normal, endotoxemia (n = 12) and 4) neutropenic and endotoxemia (n = 12).

### Neutropenia

Three days prior to study, rats received 100 mg/kg of cyclophosphamide (Sigma, USA) by intraperitoneal (IP) injection. Control rats received an equal volume of 0.9% sodium chloride. Neutropenia was confirmed by absolute neutrophil counts of less than 1000/μl.

### Endotoxin administration

Rats in the endotoxemia group were given *E. Coli *0127:B8 lipopolysaccharide (10 mg/kg) by IP injection (Sigma, USA). Control rats received an equal volume of 0.9% sodium chloride.

### Experimental protocol

Four hours after endotoxin or saline, the rats were sedated and anesthetized with ketamine (60 mg/kg) and xylazine (5 mg/kg). The right femoral artery was catheterized and mean arterial blood pressure and heart rate were recorded. Blood was removed for blood gases (Radiometer, ABL5, Westlake, OH) TNF-α, IL-1β and MIP-2. The rats were sacrificed. The right hilum was clamped and the right lower lobe of the lung was removed and placed in TRI Reagent, a RNA isolation reagent. The right ventricle was cannulated and the pulmonary circulation of the left lung was perfused with cold phosphate buffered saline until clear. The left lung was washed with 25 ml of warm (37°C) phosphate buffered saline. The bronchoalveolar lavage (BAL) fluid was centrifuged at 1200 rpm at 4°C for 7 minutes. The supernatant was stored at -70°C until analyzed.

### TNF-α, IL-1β and MIP-2 protein determination

TNF-α, IL-1β and MIP-2 were measured using a commercially available enzyme linked immunosorbent assay (ELISA) (Biosource International Inc, Camarillo, CA). In brief, plasma, BAL or known control samples were placed in a 96 well microtiter plate previously coated with monoclonal antibodies to TNF-α, IL-1β or MIP-2 respectively. A second biotinylated antibody, was added to bind to the complex followed by an enzyme horseradish peroxidase, thus forming a four-member sandwich. After addition of a substrate solution, the bound enzyme acts to produce color. The optical density was measured in a spectrophotometer at 450 nm. Concentrations of TNF-α, IL-1β or MIP-2 were determined by interpolation from the standard curve. The sensitivities for TNF-α, IL-1β and MIP-2 were <4 pg/ml, <1 pg/ml and <1 pg/ml respectively.

### TNF-α, IL-1β, and MIP-2 mRNA determination

Lung tissue mRNA was extracted as recommended by the manufacturer (Sigma, St. Louis, MO). In brief, after the lung tissue was homogenized, chloroform was added and the mixture was centrifuged. Absolute ethanol was added to the aqueous phase. After centrifugation, 75% ethanol was added to the RNA pellet. Again, the sample was centrifuged, the supernatant discarded, and the RNA pellet was placed in DEPC water. The sample was stored at -20°C until analysis.

Aliquots of the total RNA (30 μg) were resolved by formaldehyde-agarose gel electrophoresis and integrity and concentration of RNA was verified by staining with ethidium bromide. Reverse transcription of RNA (0.0625 mcg) was accomplished by (25°C for 10 minutes followed by 42°C for 60 minutes) using 5× buffer (Gibco BRL), 250 mM KCl, random primers (18 OD units), RNase inhibitor and AMV reverse transcriptase enzyme (Gibco, BRL). The RNA was then amplified by polymerase chain reaction (PCR).

Optimal conditions for TNF-α, IL-1β, MIP-2, and β-actin mRNA were established in our laboratory prior to study. Amplification was performed in 50 μl reaction buffer containing 20 mM TRIS pH 8.0, 50 mM KCl, 25 mM MgCl_2_, Taq DNA polymerase enzyme (Gibco, BRL), 2.5 mM each of dNTPs (dTTP, dGTP, dATP), 0.3 microCi dCTP radio labeled with P^32 ^and 2 μl of TNF-α, IL-1β, MIP-2, or β-actin primers (Biosource International, Camarillo, CA) respectively. The primers spanned an intron so that genomic DNA contamination would not interfere with the analyses. After denaturation for 90 sec, the thermo cycling conditions were 30 sec at 94°C, 45 sec at 72°C, 45 sec at 72°C followed by 7 min extension step at 72°C. PCR was performed with 26 cycles for TNF-α, IL-1β, and MIP-2 mRNA respectively and 21 cycles for β-actin.

A polyacrylamide gel electrophoresis (4% AA in 1% TBE buffer) was used to separate the amplified cDNA fragments. TNF-α, IL-1β, MIP-2, and β-actin mRNA were detected after exposing the gel to radiographic film. The density of the autoradiograph bands was measured using computer software (Kodak Science 1D). Relative TNF-α, IL-1β, or MIP-2 mRNA levels were estimated as the ratio of the autoradiographic density of TNF-α, IL-1β, or MIP-2 mRNA to the internal standard, β-actin mRNA.

### Statistical analysis

The values are expressed as mean ± standard deviation. The factors were compared using one-way ANOVA. A p value of < 0.05 was considered significant. If the one-way ANOVA was significant, a post hoc analysis using Games-Howell was used to determine the significance between the groups. Statistical analyses were performed using SPSS 11.5 software.

## Results

### Cardiovascular

All 4 groups had similar heart rate and mean blood pressure (table 1).

**Table 1:** Hemodynamic and Arterial Blood Gases Data

	Control	Neutropenic	LPS	Neutropenic LPS
Heart rate (bpm)	240 ± 44	238 ± 16	280 ± 38	258 ± 42
Mean blood pressure (mm Hg)	100 ± 18	107 ± 10	113 ± 20	104 ± 18
pH	7.37 ± .03	7.39 ± .09	7.29 ± .03	7.32 ± .07
pCO_2 _(torr)	47 ± 6	45 ± 11	48 ± 5	46 ± 4
pO_2_(torr)	92 ± 6	84 ± 4	80 ± 6*	69 ± 6 **
A-a O_2 _gradient (torr)	2 ± 2	8 ± 7	12 ± 9*	24 ± 5 **

*Group is different than control group, ** Group is different than all other groups p < 0.05, one-way ANOVA, post hoc test Games-Howell

### Respiratory

The pO_2 _was higher in the control and neutropenic when compared to the endotoxemic groups. However, among the endotoxemic rats, the neutropenic group had a lower pO_2 _compared to the non-neutropenic group. Likewise, the A-a O_2 _gradient was lower in the control and neutropenic when compared to the endotoxemic groups. The A-a O_2 _gradient was higher in the neutropenic, endotoxemic compared to the non-neutropenic, endotoxemic group. The pH and pCO_2 _were similar in all groups (table 1).

### Inflammation

**Figure 1:**
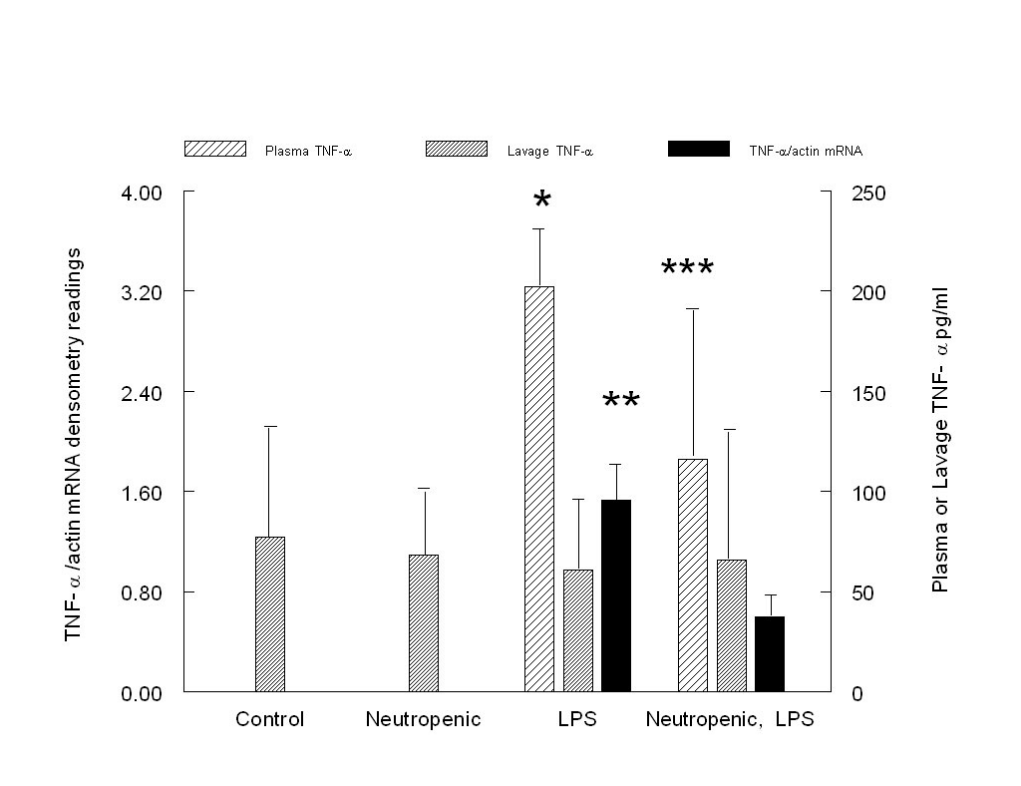
**Comparison of lung mRNA, plasma and lavage concentrations of TNF-α in the control, neutropenic, non-neutropenic, LPS and neutropenic, LPS groups**. * Plasma TNF-α is higher in normal, endotoxemic rats compared to all other groups, ** Endotoxemic group is different than the other 3 groups for lung mRNA TNF-α, *** Plasma TNF-α is higher in neutropenic, endotoxemic rats compared to control and neutropenic groups, p < 0.05, one-way ANOVA, post hoc test Games-Howell.

Neutropenic, endotoxemic rats had less pulmonary mRNA for TNF-α when compared to normal, endotoxemic rats. No mRNA for TNF-α was detected in the lungs of the control or neutropenic rats (figure 1). Likewise, among endotoxemic rats, the plasma TNF-α concentration was less in the neutropenic compared to the normal group. No plasma TNF-α was detected in either the control or neutropenic groups (figure 1). The TNF BAL concentration was similar in all four groups (77 ± 40 pg/ml for control, 68 ± 33 pg/ml for neutropenic, 61 ± 35 pg/ml for LPS, and 81 ± 54 pg/ml for neutropenic, LPS).

**Figure 2:**
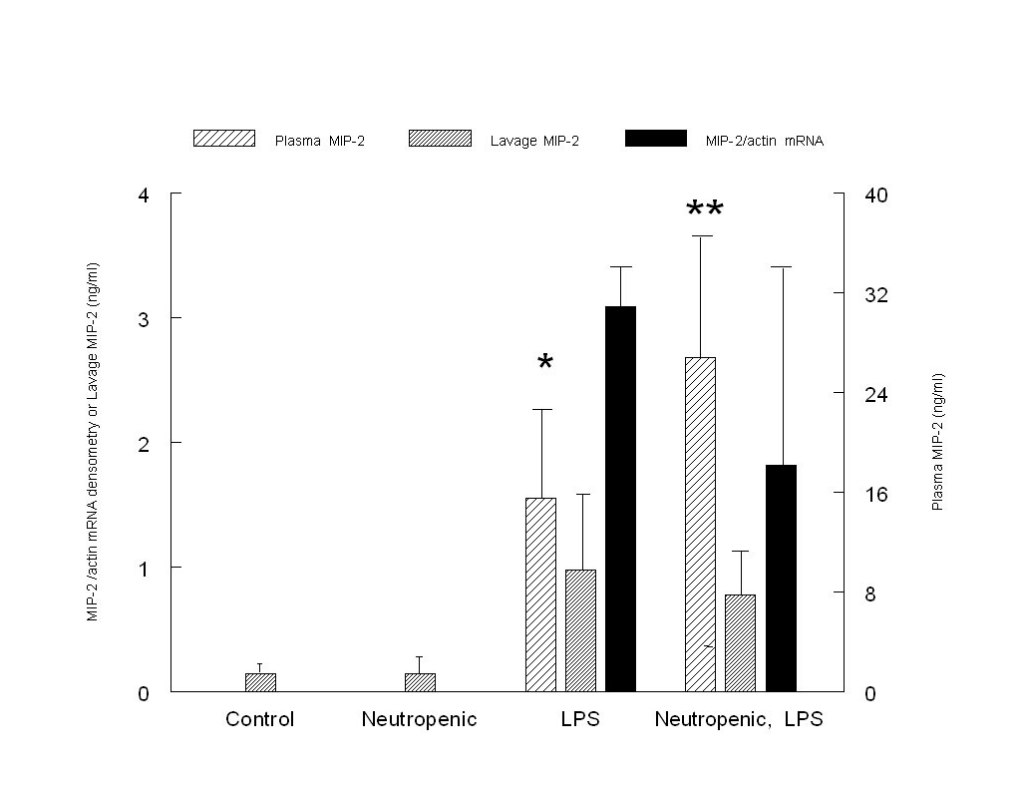
**Comparison of mRNA, plasma and lavage concentrations of MIP-2 in the control, neutropenic, endotoxemic and neutropenic, endotoxemic groups**. * Plasma MIP-2 is higher in endotoxemic rats compared to control and neutropenic groups but lower than neutropenic, endotoxemic rats, ** Plasma MIP-2 is higher in the endotoxemic, neutropenic group compared to all other groups, p < 0.05, one-way ANOVA, post hoc test Games-Howell.

Plasma concentrations of MIP-2 were elevated in the endotoxemic, neutropenic rats when compared to the endotoxemic, normal rats (figure 2). However, pulmonary mRNA for MIP-2 was similar in both endotoxemic groups. In control and neutropenic rats, no mRNA for MIP-2 was detected in the lung. Lung lavage concentrations of MIP-2 were higher in the endotoxemic rats compared to the control and neutropenic groups. However, the MIP-2 lung lavage concentrations were similar in the endotoxemic groups regardless of the presence or absence of neutropenia (figure 2).

Endotoxemic, neutropenic rats had similar amounts of mRNA for IL-1β in the lung compared to endotoxemic, normal rats (0.51 ± 0.19 vs. 0.87 ± 0.43, densitometry readings IL-1β mRNA/β-actin mRNA, post hoc analysis, p = 0.32). The control and neutropenic rats had no detection of mRNA for IL-1β (one-way ANOVA, p < 0.05 for endotoxemic vs. non-endotoxemic rats). IL-1β was not detected in the plasma or lavage of any of the groups.

## Discussion

The early effect of endotoxin on cytokine production in the systemic circulation has not been well studied in the neutropenic host. We compared the early production of TNF-α, IL-1β, and MIP-2 in the systemic circulation of neutropenic and non-neutropenic rats after endotoxin administration. In this model of endotoxemia, the TNF-α concentration was markedly decreased while MIP-2 was elevated in the plasma of the neutropenic, endotoxemic compared to non-neutropenic, endotoxemic rats. IL-1β was not detected in either group. Previous studies have shown that the early response to endotoxin would be an elevation in TNF-α and IL-1β followed by a rise in MIP-2 production [23,24]. Thus, it appears that the neutrophil is involved directly or indirectly in increasing TNF-α production in the systemic circulation after endotoxemia. Neutrophils may effect their own recruitment into the systemic circulation by down-regulating the production of the chemokine MIP-2. In this model of early endotoxemia, IL-1β production is not yet present in the systemic circulation. In endotoxemia, cytokines from the systemic circulation can increase the permeability of the endothelium of the alveolar capillaries thus precipitating acute lung injury [25].

In our study, acute lung injury as shown by increased A-a O_2 _gradient developed within 4 hours of the administration of endotoxin in both neutropenic and non-neutropenic rats. The neutropenic rats had evidence of more severe lung injury when compared to the non-neutropenic rats. Tumor necrosis factor-α plays an important role in initiating acute lung injury after endotoxemia in the normal host [26,27]. However, the influence of neutrophils on the production of TNF-α is controversial [6,28-32]. In an endotoxemia model, pulmonary mRNA and protein concentration of TNF-α were similar in both neutropenic and non-neutropenic mice. Alternatively, in the same study, elevations in pulmonary mRNA and protein concentration of TNF-α were observed in a neutropenic compared to non-neutropenic mouse hemorrhagic shock model [6]. In a study where pulmonary TNF-α mRNA and TNF-α in lung homogenates were compared in neutropenic rats given granulocyte colony stimulating factor (GCSF) for recovery versus placebo, the GCSF group had higher amounts of TNF-α mRNA and TNF-α in lung homogenates [33]. This suggests that the neutrophils play a role in causing a rise in TNF-α either by producing TNF-α or influencing other cells to produce it. In our study, endotoxemia did not lead to the TNF-α production in the lung. However, the mRNA for TNF-α was decreased in the lungs of the neutropenic, endotoxemic versus the non-neutropenic, endotoxemic rats. This suggests that the presence of neutrophils may alter the production of TNF-α in the lung which then may influence the inflammatory response later in the course of endotoxemia. The observed differences between studies may be a function of timing. Measurements of TNF-α at numerous time points may be beneficial.

In addition to TNF-α, IL-1β may play a significant role in the development of acute lung injury [15]. Pulmonary mRNA and protein for IL-1β, one of the earliest inflammatory cytokines, has been shown to be suppressed in neutropenic mice who received endotoxin by intraperitoneal injection [6]. In neutropenic rats that received GCSF prior to acute lung injury, the lung homogenate IL-1β concentration was much higher when compared to those neutropenic rats that did not receive GCSF suggesting that the increased number of neutrophils was responsible for IL-1β production [33]. In our study, neutropenic rats tended to have decreased amounts of pulmonary IL-1β mRNA when compared to non-neutropenic, endotoxemic rats; however the difference was not significant. IL-1β was not detected in the lung lavage. It is possible that in this early model of endotoxemia, IL-1β was not yet produced.

Endotoxemia affected the production of MIP-2 in the lavage and mRNA for MIP-2 in the lung when compared to control rats. However, the concentration of MIP-2 in the lavage and the detection of mRNA for MIP-2 were similar in both the neutropenic and non-neutropenic groups. It is likely that neutrophil chemotaxis has not yet occurred and therefore feedback inhibition by the neutrophil has not transpired at this time point. PMN staining would provide evidence for chemotaxis/no-chemotaxis at this time point.

In spite of the fact that the cytokine production in the lung was similar in both endotoxemic groups, acute lung injury was more severe in the neutropenic compared to the non-neutropenic, endotoxemic rat. An increase in pulmonary vascular resistance or a decline in arterial oxygenation are the first indicators of acute lung injury [34]. Early in acute lung injury, low oxygenation can be the result of ventilation perfusion mismatch [25]. This can develop as a result of an imbalance of the effect of vasodilators and vasoconstrictors on the pulmonary vascular endothelium [34-36]. In addition, some studies suggest that a decreased endothelium-dependent relaxation and increased constrictor response results in spite of expression of inducible nitric oxide synthase and the release of nitric oxide [37-39]. As lung injury progresses, pulmonary edema which develops in part due to cytokines, plays a role in decline in oxygenation [25]. In our study, in the absence of cytokine production in the lung, it is possible that pulmonary hypertension due to an imbalance of vasodilators and vasoconstrictors or impaired sensitivity to vasoactive mediators is the cause of the low oxygenation. Further investigation into the cause of this early deterioration in lung function is warranted.

## Conclusion

Neutropenia is associated with the production or regulation of TNF-α in endotoxemia. Likewise, neutrophils may influence their own chemotaxis by regulating MIP-2 production in endotoxemia. However, neutrophils may act indirectly by regulating cytokine production in other inflammatory cells. Further investigation is required to determine how the neutrophil influences the inflammatory process in sepsis.

In early endotoxemia, the more severe lung injury observed in neutropenic compared to non-neutropenic rats does not depend on TNF-α, IL-1β and MIP-2 in the lung. In fact, the neutrophil may be responsible for indirectly injuring the lung by its influence on the macrophage or endothelial cell such as in nitric oxide production.

## References

[B1] Anderson MR, Blumer JL (1997). Advances in the therapy for sepsis in children. Pediatr Clin North Am.

[B2] Romano V, Castagnola E, Dallorso S, Lanino E, Calvi A, Silvestro S, Morreale G, Giacchino R, Dini G (1999). Bloodstream infections can develop late (after day 100) and/or in the absence of neutropenia in children receiving allogeneic bone marrow transplantation. Bone Marrow Transplant.

[B3] Ben-Hur H, Kossoy G, Kossoy N, Zusman I (2002). Response of the immune system of mammary tumor-bearing rats to cyclophosphamide and soluble low-molecular-mass tumor-associated antigens: the bone marrow and thymus. Int J Mol Med.

[B4] Tanaka H, Miyazaki S, Sumiyama Y, Kakiuchi T (2004). Role of macrophages in a mouse model of postoperative MRSA enteritis. J Surg Res.

[B5] Karni A, Balashov K, Hancock WW, Bharanidharan P, Abraham M, Khoury SJ, Weiner HL (2004). Cyclophosphamide modulates CD4+ T cells into a T helper type 2 phenotype and reverses increased IFN-gamma production of CD8+ T cells in secondary progressive multiple sclerosis. J Neuroimmunol.

[B6] Abraham E, Carmody A, Shenkar R, Arcaroli J (2000). Neutrophils as early immunologic effectors in hemorrhage- or endotoxemia-induced acute lung injury. Am J Physiol Lung Cell Mol Physiol.

[B7] Bhatia M, Moochhala S (2004). Role of inflammatory mediators in the pathophysiology of acute respiratory distress syndrome. J Pathol.

[B8] Martin TR (1999). Lung cytokines and ARDS: Roger S. Mitchell Lecture. Chest.

[B9] Strieter RM, Kunkel SL, Keane MP, Standiford TJ (1999). Chemokines in lung injury: Thomas A. Neff Lecture. Chest.

[B10] Driscoll KE (1994). Macrophage inflammatory proteins: biology and role in pulmonary inflammation. Exp Lung Res.

[B11] Matsushima K, Oppenheim JJ (1989). Interleukin 8 and MCAF: novel inflammatory cytokines inducible by IL 1 and TNF. Cytokine.

[B12] Oppenheim JJ, Zachariae CO, Mukaida N, Matsushima K (1991). Properties of the novel proinflammatory supergene "intercrine" cytokine family. Annu Rev Immunol.

[B13] Duffy AJ, Nolan B, Sheth K, Collette H, De M, Bankey PE (2000). Inhibition of alveolar neutrophil immigration in endotoxemia is macrophage inflammatory protein 2 independent. J Surg Res.

[B14] Hudson LD, Milberg JA, Anardi D, Maunder RJ (1995). Clinical risks for development of the acute respiratory distress syndrome. Am J Respir Crit Care Med.

[B15] Goodman RB, Pugin J, Lee JS, Matthay MA (2003). Cytokine-mediated inflammation in acute lung injury. Cytokine Growth Factor Rev.

[B16] Schmal H, Shanley TP, Jones ML, Friedl HP, Ward PA (1996). Role for macrophage inflammatory protein-2 in lipopolysaccharide-induced lung injury in rats. J Immunol.

[B17] Gupta S, Feng L, Yoshimura T, Redick J, Fu SM, Rose CE (1996). Intra-alveolar macrophage-inflammatory peptide 2 induces rapid neutrophil localization in the lung. Am J Respir Cell Mol Biol.

[B18] Huang S, Paulauskis JD, Godleski JJ, Kobzik L (1992). Expression of macrophage inflammatory protein-2 and KC mRNA in pulmonary inflammation. Am J Pathol.

[B19] Mercer-Jones MA, Heinzelmann M, Peyton JC, Wickel DJ, Cook M, Cheadle WG (1997). The pulmonary inflammatory response to experimental fecal peritonitis: relative roles of tumor necrosis factor-alpha and endotoxin. Inflammation.

[B20] Driscoll KE, Hassenbein DG, Howard BW, Isfort RJ, Cody D, Tindal MH, Suchanek M, Carter JM (1995). Cloning, expression, and functional characterization of rat MIP-2: a neutrophil chemoattractant and epithelial cell mitogen. J Leukoc Biol.

[B21] Rose CE, Juliano CA, Tracey DE, Yoshimura T, Fu SM (1994). Role of interleukin-1 in endotoxin-induced lung injury in the rat. Am J Respir Cell Mol Biol.

[B22] Xing Z, Jordana M, Kirpalani H, Driscoll KE, Schall TJ, Gauldie J (1994). Cytokine expression by neutrophils and macrophages in vivo: endotoxin induces tumor necrosis factor-alpha, macrophage inflammatory protein-2, interleukin-1 beta, and interleukin-6 but not RANTES or transforming growth factor-beta 1 mRNA expression in acute lung inflammation. Am J Respir Cell Mol Biol.

[B23] Xu WB, Haddad EB, Tsukagoshi H, Adcock I, Barnes PJ, Chung KF (1995). Induction of macrophage inflammatory protein 2 gene expression by interleukin 1 beta in rat lung. Thorax.

[B24] Xavier AM, Isowa N, Cai L, Dziak E, Opas M, McRitchie DI, Slutsky AS, Keshavjee SH, Liu M (1999). Tumor necrosis factor-alpha mediates lipopolysaccharide-induced macrophage inflammatory protein-2 release from alveolar epithelial cells. Autoregulation in host defense. Am J Respir Cell Mol Biol.

[B25] Groeneveld AB (2002). Vascular pharmacology of acute lung injury and acute respiratory distress syndrome. Vascul Pharmacol.

[B26] Tracey KJ, Lowry SF, Cerami A (1988). Cachetin/TNF-alpha in septic shock and septic adult respiratory distress syndrome. Am Rev Respir Dis.

[B27] Li XY, Donaldson K, Brown D, MacNee W (1995). The role of tumor necrosis factor in increased airspace epithelial permeability in acute lung inflammation. Am J Respir Cell Mol Biol.

[B28] Abraham E, Kaneko DJ, Shenkar R (1999). Effects of endogenous and exogenous catecholamines on LPS-induced neutrophil trafficking and activation. Am J Physiol.

[B29] Cassatella MA (1995). The production of cytokines by polymorphonuclear neutrophils. Immunol Today.

[B30] Parsey MV, Tuder RM, Abraham E (1998). Neutrophils are major contributors to intraparenchymal lung IL-1 beta expression after hemorrhage and endotoxemia. J Immunol.

[B31] Shenkar R, Abraham E (1999). Mechanisms of lung neutrophil activation after hemorrhage or endotoxemia: roles of reactive oxygen intermediates, NF-kappa B, and cyclic AMP response element binding protein. J Immunol.

[B32] Pugin J, Ricou B, Steinberg KP, Suter PM, Martin TR (1996). Proinflammatory activity in bronchoalveolar lavage fluids from patients with ARDS, a prominent role for interleukin-1. Am J Respir Crit Care Med.

[B33] Azoulay E, Attalah H, Yang K, Herigault S, Jouault H, Brun-Buisson C, Brochard L, Harf A, Schlemmer B, Delclaux C (2003). Exacerbation with granulocyte colony-stimulating factor of prior acute lung injury during neutropenia recovery in rats. Crit Care Med.

[B34] Nakazawa H, Noda H, Noshima S, Flynn JT, Traber LD, Herndon DN, Traber DL (1993). Pulmonary transvascular fluid flux and cardiovascular function in sheep with chronic sepsis. J Appl Physiol.

[B35] Hales CA, Sonne L, Peterson M, Kong D, Miller M, Watkins WD (1981). Role of thromboxane and prostacyclin in pulmonary vasomotor changes after endotoxin in dogs. J Clin Invest.

[B36] Ichinose F, Zapol WM, Sapirstein A, Ullrich R, Tager AM, Coggins K, Jones R, Bloch KD (2001). Attenuation of hypoxic pulmonary vasoconstriction by endotoxemia requires 5-lipoxygenase in mice. Circ Res.

[B37] Griffiths MJ, Curzen NP, Mitchell JA, Evans TW (1997). In vivo treatment with endotoxin increases rat pulmonary vascular contractility despite NOS induction. Am J Respir Crit Care Med.

[B38] Terraz S, Baechtold F, Renard D, Barsi A, Rosselet A, Gnaegi A, Liaudet L, Lazor R, Haefliger JA, Schaad N, Perret C, Kucera P, Markert M, Feihl F (1999). Hypoxic contraction of small pulmonary arteries from normal and endotoxemic rats: fundamental role of NO. Am J Physiol.

[B39] Pulido EJ, Shames BD, Fullerton DA, Sheridan BC, Selzman CH, Gamboni-Robertson F, Bensard DD, McIntyre RC (2000). Differential inducible nitric oxide synthase expression in systemic and pulmonary vessels after endotoxin. Am J Physiol Regul Integr Comp Physiol.

